# Tropical Hæmatology.—III

**Published:** 1910-04-02

**Authors:** 


### Pathology.

### TROPICAL HÆMATOLOGY.—III.

(*Continued from page 544.*)

*The method of counting the blood by fields* (Wright's method).—To some this method is simpler than that of the hæmocytometer. The results come out very much the same, especially so if two individuals use the two methods side by side. It is carried out as follows: (1) Mark on the draw-tube of your microscope (using 2-eyepiece and $\frac{1}{6}''$ objective) the amount drawn out which will give a field having the diameter of 8 Thoma Zeiss squares; each square equals $\frac{1}{20}$ of a mm. across. (2) Make dilution of blood 1 in 20 for whites (Thoma Zeiss), 1 in 10,000 for reds (1-200 (Thoma Zeiss), blow this into watch-glass, one of this to 10 of diluting fluid gives dilution of 1-2000, one of this to 5 of diluting fluid gives 1-10000). (3) Place drop on counting plate and cover; enumerate total number of cells in 20 fields; take average. *Calculation.*—Multiply this by cubic capacity of each field = $\frac{1}{80}$ c.mm., and by dilution; this gives the number of corpuscles per c.mm. For example, 5 whites per field × 80 × 20 = 8000 per c.mm. Rationale.—To convert square into circle × by $PR^2$. $P = 3\frac{1}{7}$. Depth of cell = $\frac{1}{10}$ (standard) mm. Width of field = 0.4 (1 sq. = .05 × 8 = 0.4), ∴ radius = .2 or $\frac{1}{5}$ mm. Then $\frac{22}{7} \times \frac{1}{5}^2 = \frac{22}{175}$, or nearly $\frac{1}{8}$; this × by $\frac{1}{10} = \frac{1}{80}$, or the cubic capacity of each field.

*The Hæmatocrit of Hedin.*—This apparatus is often supplied with one of the small centrifuges at present on the market. It consists of a flat metal band, easily fixable to the centrifuge, and into which are let two capillary glass tubes (one at each end).

*Technique.*—Take one of the capillary tubes off and, by means of a piece of rubber tubing (one of the Thoma Zeiss rubber tubes will do) suck up blood into it so as entirely to fill it. As soon as full place finger greased with vaseline over its free end, and then draw off the rubber piping. Adjust it back again in its place and centrifuge vigorously for three minutes or more. The red corpuscles separate from the serum, the white cells accumulating as a greyish blur at the end of the column. If one examine the tube now one notices that it is divided into 100 equal divisions, each degree on the scale being called 100,000 cells or a little more. Suppose, after vigorous centrifuging, the end of the red column stands at 40, that means the number of red blood corpuscles per c.mm. is a little over 4,000,000, or, if at 25, a little over 2,500,000, and so on. The method is inapplicable in cases of severe anæmia, leukemia, etc., though for getting a rough idea of the quality of the blood in practice it is useful enough.

2. *The estimation of the hæmoglobin.*—This is done by instruments called hæmometers or hæmoglobinometers, the principle being the comparison of diluted blood with standard colour solutions (gelatin, glass, paper, etc.). Many are on the market, the better known being as follows: 1. *Gower's hæmoglobinometer.* This consists of two tubes, a pipette, and a bottle for distilled water. The standard tube consists of coloured gelatin, the other (the empty one) being graduated in percentages. Suck up 20 c.mm. of blood in the pipette (pipette is marked at this point), wipe off all blood adhering to outside, then blow this into empty tube, into which a drop or two of distilled water has been added. Go on now adding more distilled water till the colour in tube approximates to that of standard. Test by holding the two tubes up together alternately to the light alone, and then to the light with a piece of white paper as background, and when colours are the same read off percentage on tube. This gives percentage of hæmoglobin. 2. *Oliver's hæmoglobinometer.* One here fills the capillary pipette supplied with the instrument with blood, this being washed out with distilled water into the mixing cell, which is then filled to the brim with distilled water. A small glass plate is then applied and the colour is compared with a series of standard coloured discs. When approximation takes place the number of the standard disc gives the percentage of hæmoglobin. Glass riders have to be used to get the decimals, and different sets of discs are required for artificial light if that is used. The daylight ones are said to be less accurate than the artificial ones, so the latter should be employed.

3. *Von Fleischl's hæmometer.* Though more expensive than some of the other hæmoglobinometers (£3), this instrument is said to be extremely accurate and, therefore, is largely used. A dark room where artificial light can be employed is necessary, and this is a disadvantage in some ways. The advantages claimed for it are: (1) that it is very easy and convenient to use; (2) that the amount of hæmoglobin can be quickly and accurately read in percentages; and (3) that the amount of blood required is very small (only a drop). The instrument consists of a stout platform or stand with a cell divided into two equal parts fitting on to this, while underneath the stand a graduated coloured glass slab is fixed, this being capable of being run backwards or forwards by means of a screw. The blood is drawn up into small capillary tubes, and it must be remembered that when ordering extra tubes the number stamped on the metal holder must be given, as each instrument is calibrated to suit capillary tubes of a definite capacity. Procedure for use: Fill a capillary tube with blood; blow this into one of the chambers which has been filled with distilled water; fill the other chamber with distilled water alone; arrange on stand, latter being over graduated glass slab; move screw till two colours correspond, focussing light by means of mirror below stand; then read off percentage, this being indicated by a mark on instrument. Cabot gives the further precautions for use: (1) The capillary tube must be clean and dry, otherwise the blood will not run up. (2) Be careful that neither fluid intermingles.

(3) Stand sideways so as to get the compartments whose colours we are to match on the right and left halves of the retina, which are equally sensitive to light (the upper half of the retina is less sensitive than the lower). (4) Use as little light as possible. (5) Use the black tube (supplied with instrument) to look through. (6) Use first one eye then the other, and never look more than a few seconds at a time. (7) Move the thumb-screw with short, quick turns. (8) If the percentage of hæmoglobin is 30 per cent. or less, two or three pipettes of blood should be used and the reading divided by 2 or 3. Considerable error may thus be avoided.

4. *Fleischl-Miescher's hæmometer.*—This is said to be a further improvement on the former. It is more expensive (£5 13s.). Both are patented by Reichert, of Vienna.

5. *Talquist's scale.*—A drop of blood is caught on a piece of clean filter-paper; it is then allowed to dry and is at once compared with standard tinted papers. These having a circular hole cut in their middle, the filter-paper can be quickly passed behind them. It only indicates the tens; for rough clinical use it is fairly accurate.

6. *Dare's hæmoglobinometer.*—The principle of this instrument is that no dilution of blood is required. A thin layer of blood is run into a cell, this being then examined by transmitted light and compared with standard. It is convenient and said to be accurate.

